# Evolution of overlying strata and disaster-causing mechanisms under inclined thin coal seam group mining: insights from numerical simulations

**DOI:** 10.1038/s41598-026-56411-x

**Published:** 2026-06-12

**Authors:** Yingxi Li, Fengyan Zhang

**Affiliations:** 1 College of Mining, Liaoning Technical University, Fuxin, 123000 Liaoning China; 2 College of Mine Safety, North China Institute of Science and Technology, Langfang, 065201 Hebei China

**Keywords:** Inclined thin coal seam group, Numerical simulation, Overlying strata, Hazard-causing mechanisms, Energy science and technology, Engineering, Natural hazards, Solid Earth sciences

## Abstract

To clarify the overburden evolution and asymmetric instability mechanism during bottom-up repeated mining of an inclined thin coal seam group, a 3DEC numerical model was established based on geological conditions in Guizhou Province. The caving behavior, stress redistribution, displacement response, and simulated fracture-trace complexity of the overlying strata were analyzed during the first mining (M1), second mining (M2), and third mining (M3) stages. The results show that M1 mining mainly induces local subsidence, goaf compaction, and limited fracture development. With the successive extraction of M2 and M3, interlayer disturbance is progressively superimposed, the overburden failure range expands upward, and the stress field evolves into a zoned pattern characterized by stress relief in the goaf and stress concentration near the working face and goaf boundaries. The displacement field also shows a cumulative response, with the high-displacement zone migrating upward and the subsidence trough becoming deeper and wider. Under the 30° inclined structure, overburden deformation and failure exhibit clear dip-direction asymmetry: the high side is dominated by separation, sliding, and fracture development, whereas the low side mainly shows compaction and constrained deformation. The apparent fractal dimension of simulated fracture traces increases from 1.023 at M1 to 1.096 at M2 and 1.144 at M3, indicating enhanced relative complexity under identical discretization conditions. The overburden hazard-causing mechanism is therefore interpreted as a coupled process of stress redistribution, uncoordinated subsidence, high-side fracture development, and asymmetric instability of the load-bearing structure. These findings provide guidance for roof stability assessment and hazard control in inclined thin coal seam group mining.

## Introduction

Coal, as China’s primary energy source and an important industrial raw material, has long played a fundamental role in safeguarding national energy security and supporting socioeconomic development^1–4^. China’s coal resources occur under complex geological conditions^5–9^, and coal seam groups, particularly inclined thin coal seam groups, are widely distributed and serve as important replacement resources for maintaining stable and high mine production^10–14^. Compared with near-horizontal single-seam mining, the mining of inclined thin coal seam groups is characterized by large seam dip angles, strong interlayer disturbance, complex structural responses of the overlying strata, and pronounced superimposed effects induced by repeated mining^15–18^. During bottom-up sequential mining, mining-induced disturbance not only alters the stress state and movement patterns of the overlying strata but may also trigger a series of engineering problems^19–22^, including overlying strata caving, key strata instability, fracture coalescence, and surrounding rock hazards in the mining field^23–26^. In particular, under inclined structural conditions, the gravitational component acting along the dip direction causes markedly spatially non-uniform deformation and failure of the overlying strata^27–29^, thereby further increasing the difficulty of roof hazard control and safe mining. Therefore, systematically revealing the evolution of caving, stress, displacement, and fractures in the overlying strata during repeated mining of inclined thin coal seam groups, and clarifying the associated asymmetric instability-induced hazard-causing mechanisms, are of great theoretical significance and engineering value for improving the mining theory of inclined coal seam groups, guiding roof hazard prevention and control, and ensuring safe and efficient mine extraction.

In recent years, extensive studies have been conducted worldwide on the evolution of overlying strata and hazard control during coal seam mining^30–33^, leading to substantial progress in understanding overlying strata caving behavior, stress redistribution, rock mass movement and deformation, and mining-induced fracture development^34–39^. Existing studies have shown that mining activities can generate a pressure-relief zone above the goaf and concentrated loading zones on both sides, while overlying strata caving and fracture development exhibit pronounced stage-dependent characteristics^40–43^. Meanwhile, rock stratum movement, key strata breakage, and fracture propagation are important factors controlling mining-field stability and hazard evolution^44–46^. With respect to inclined coal seam mining, some studies have examined the influence of seam dip angle on roof movement, stress distribution, and strata pressure behavior, indicating that inclined structures can induce a certain degree of asymmetry in overlying strata deformation and failure^47–51^. For example, Liu et al.^52^ found through numerical modelling that, after mining in an inclined coal seam, an arch-shaped stress-relief zone forms above the goaf and is asymmetrically distributed along the dip direction. Hou et al.^53^ investigated gas migration and permeability evolution during in situ stress release induced by mining using experiments and numerical simulation. Zhang et al.^54^ examined the collapse characteristics and subsidence behavior of overlying strata during repeated extraction in gently inclined coal seams, and further analysed the spatial distribution and transfer mechanism of the mining stress field through numerical simulation. Zhao et al.^55^ carried out two-dimensional physical similarity simulation experiments under different seam dip conditions, revealing the fracture mechanical characteristics and energy evolution patterns of the rock mass at different dip angles.

However, existing studies have mainly focused on single-seam mining conditions, and the evolution of overlying strata under bottom-up repeated mining of inclined thin coal seam groups remains insufficiently understood. In particular, the intrinsic relationships among overlying strata caving, stress redistribution, displacement development, and fracture network complexification under repeated multi-seam mining have not yet been systematically clarified. Moreover, the hazard-causing chain linking asymmetric evolution, structural instability, and hazard formation of the overlying strata under the control of inclined structures requires further investigation.

Accordingly, this study takes the bottom-up sequential mining of an inclined thin coal seam group as the research background and combines numerical simulation with fractal analysis to systematically investigate the evolution of the overlying strata and the associated hazard-causing mechanisms under repeated mining. First, the caving behavior and structural response characteristics of the overlying strata at different mining stages are analyzed. Second, the dynamic evolution of the stress and displacement fields in the overlying coal–rock mass is examined. Furthermore, based on binary fracture image processing and the box-counting dimension method, the complexity of the fracture network in the overlying strata is quantitatively characterized. Finally, by integrating the evolution characteristics of caving, stress, displacement, and fractures, an asymmetric instability-induced hazard-causing mechanism of the overlying strata during inclined thin coal seam group mining is established. The results provide a theoretical basis for roof hazard prevention and control and for safe mining under inclined thin coal seam group extraction conditions.

Compared with previous studies that mainly focused on single-seam mining or the independent analysis of stress, displacement, and roof caving, the main contribution of this study lies in establishing a coupled interpretation framework for asymmetric overlying-strata instability during repeated mining of an inclined thin coal seam group. The novelty of this work can be summarized as follows. First, the combined effects of repeated mining disturbance and seam inclination are clarified by linking overburden caving, stress redistribution, displacement evolution, and numerical contact-failure patterns. Then, a progressive hazard-causing chain is proposed. This provides a new mechanistic understanding of why roof instability and structural failure are more likely to develop asymmetrically during inclined coal seam group mining.

## Methods

### Engineering geological background

**Fig. 1:**
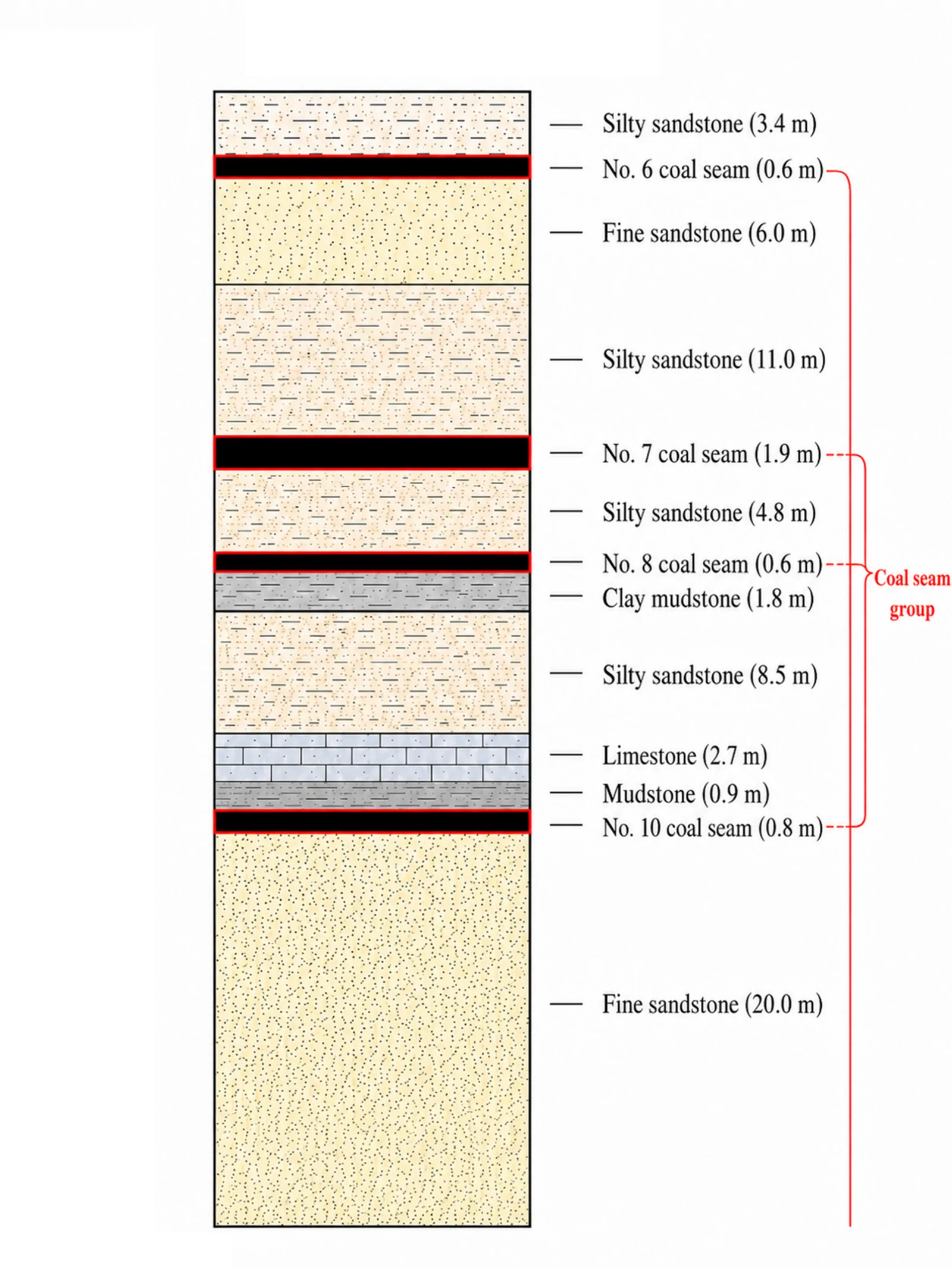
Geological profile showing the distribution of the coal seam group in the study area.

This study was conducted using a coal mine in Guizhou Province as the engineering background. The coal-bearing strata within the mine field belong to the Longtan Formation, which consists of alternating marine–continental sedimentary sequences composed of fine sandstone, siltstone, mudstone, limestone, and coal seams. The mine field is divided into two mining levels and five mining districts. The elevation of the first mining level is + 1026 m, while that of the second mining level is + 805 m. At present, mining activities are being carried out in the second district of the second mining level. This mining district contains four mineable or locally mineable coal seams, namely the No. 6, No. 7, No. 8, and No. 10 coal seams from top to bottom, with an average seam dip angle of 30°. Among them, the No. 6 and No. 8 coal seams are locally mineable. As shown in Fig. 1, the No. 10 coal seam occurs in the lower part of the coal-bearing rock series and lies 19.33–21.64 m below the No. 7 coal seam, with an average spacing of approximately 20 m. The average thicknesses of the No. 10 and No. 7 coal seams are 0.8 m and 1.93 m, respectively. In addition, the mine is classified as a coal and gas outburst mine. A combined adit–inclined shaft development system is adopted. Among the working faces, the 21,002 working face is the first mining face in the second mining district and is located in the No. 10 coal seam. The working face is 200 m long, with the transport roadway located at elevations of + 1020 to + 1031 m and the return airway at elevations of + 1080 to + 1081 m. Its roof strata are composed mainly of mudstone, limestone, siltstone, and clay mudstone.

### Numerical model design and simulation scheme

**Fig. 2:**
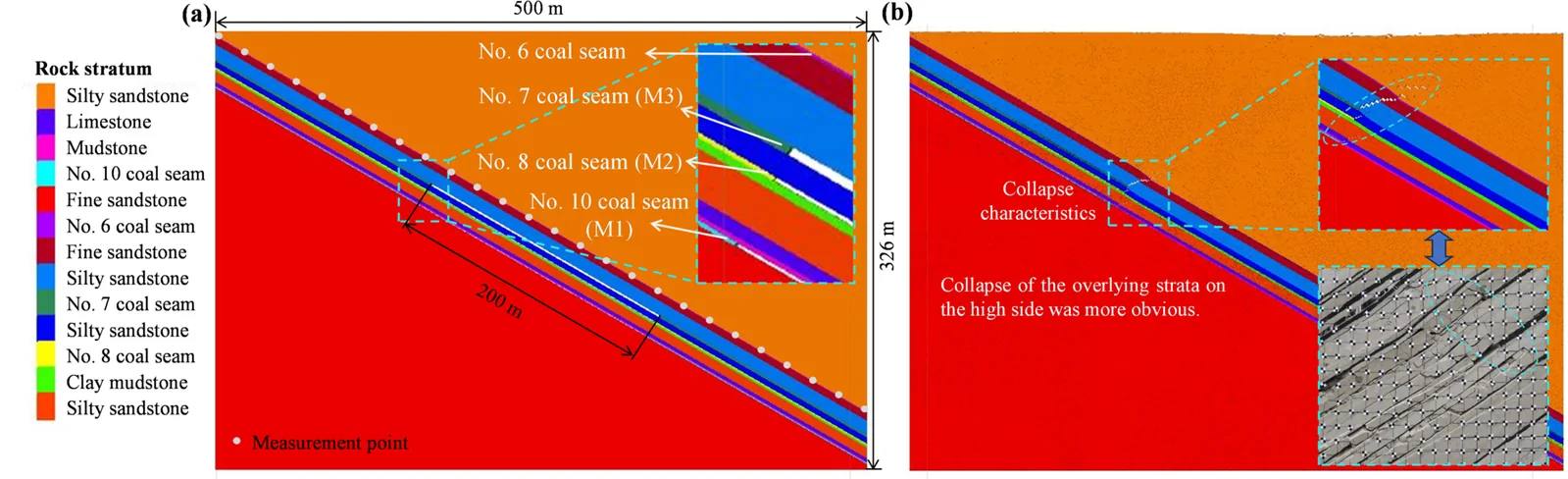
Establishment of the numerical model and demonstration of simulation results. (**a**) Numerical model and simulation scheme; (**b**) caving characteristics of the overlying strata after mining completion and comparative validation 15.

To investigate the stress evolution and displacement distribution of the overlying strata during multi-seam mining, this study established a two-dimensional inclined numerical model based on a coal mine in Guizhou Province using 3DEC to simulate the disturbance effects of mining activities on the overlying strata. The two-dimensional geometric model has a width of 500 m, a height of 326 m, and a dip angle of 30° (Fig. 2). The numerical model was established and solved using 3DEC version 5.2 developed by Itasca Consulting Group. The model was discretized into deformable blocks with a non-uniform block size. The rock strata and coal seams were simulated using the Mohr–Coulomb constitutive model, and the contacts between adjacent blocks were described using the Coulomb-slip contact model. Before each excavation step, the initial stress field was first brought to mechanical equilibrium under gravity loading and boundary constraints. Each mining step was then simulated by excavating 40 m of the corresponding coal seam, followed by sufficient calculation cycles until the model reached equilibrium again. The convergence of each calculation stage was judged according to the unbalanced force ratio and the stability of the monitoring variables. Specifically, the calculation was considered to have reached equilibrium when the average unbalanced force ratio was lower than 1 × 10⁻^5^ and the variations in displacement and stress at the monitoring points became negligible during successive calculation steps. The same convergence criterion was used for all mining stages to ensure consistency among the M1, M2, and M3 simulations. Horizontal displacement constraints were applied to the left and right boundaries, while vertical movement along the side boundaries was allowed. The bottom boundary was fixed in both the horizontal and vertical directions, and the top boundary was left free. Gravity loading and stress compensation based on the burial depth were applied to reproduce the initial in situ stress state.

The mechanical parameters listed in Table 1 were determined through a combined procedure involving geological data collection, laboratory testing, literature reference, and numerical calibration. The thicknesses of the rock strata and coal seams, the seam spacing, the seam dip angle, and the mining geometry were obtained from the geological report and mining layout of the study area. The basic mechanical parameters, including density, elastic modulus, Poisson’s ratio, cohesion, internal friction angle, and tensile strength, were mainly determined from laboratory tests on representative field rock and coal samples. For strata for which complete laboratory data were unavailable, the parameters were supplemented by referring to similar lithologies in the same mining area and relevant published studies.

Figure 2 presents a simplified schematic of the extraction sequence for the different coal seams. To simulate the effects of multi-seam mining, the coal seams were excavated step by step according to the actual engineering requirements, with the first mining No. 10 coal seam mined (M1), followed by the second mining No. 8 coal seam (M2), and finally the third mining No. 7 coal seam (M3). Each seam was extracted in five steps, with 40 m mined in each step, giving a total advance length of 200 m. After each excavation step, the stress redistribution and deformation-failure process of the overlying strata were simulated. Throughout the entire mining process, 26 monitoring points were arranged from left to right along the floor of the No. 6 coal seam to record the stress redistribution and displacement variation at different stages. Figure 2b shows a schematic of the caving pattern of the overlying strata in the model after mining was completed. Following coal seam extraction, the roof and overlying strata exhibited block-type caving over a certain scale, and the caving was more pronounced on the upper side, namely the high side along the dip direction. This demonstrates a distinct asymmetric caving characteristic.

To evaluate the rationality of the numerical model, the simulation results were compared with previously reported physical similarity simulation results under similar inclined coal seam group mining conditions 15. It should be emphasized that the purpose of this comparison was not to achieve a point-by-point quantitative agreement between the numerical model and the physical model. Because of differences in model scale, boundary conditions, material simplification, block discretization, and excavation representation, strict numerical consistency in displacement magnitude, caving height, or local failure boundaries is difficult to obtain. Therefore, the validation in this study focuses mainly on whether the numerical model can reasonably reproduce the dominant deformation and failure characteristics of the overlying strata, including the upward expansion of the disturbed zone, the progressive enlargement of the failure range, and the asymmetric evolution between the high side and the low side. The comparison shows that both the numerical simulation and the physical similarity simulation exhibit similar overall characteristics. This trend-based comparison indicates that the numerical model is suitable for analyzing the general evolution mechanism of overburden deformation, failure-range development, stress redistribution, and asymmetric instability during repeated mining of the inclined thin coal seam group.

**Table 1:** Numerical modeling physical mechanical parameters.

Rock type	Thicknesses (m)	Capacity (kg/m^3^)	Elastic modulus (GPa)	Poisson’s ratio	Cohesive force (MPa)	Internal friction angle (°)	Tensile strength (MPa)	Expansion angle (°)
Silty sandstone	3.4	2500	11.47	0.27	35.08	24.6	3.7	6
No. 6 coal seam	0.6	1400	3.07	0.21	1.02	12.3	1.32	10
Fine sandstone	6	2500	20.04	0.28	22.12	15.6	7.96	6
Silty sandstone	11	2500	11.47	0.27	35.08	24.6	3.7	6
No. 7 coal seam	1.9	1400	4.00	0.21	1.02	12.3	1.32	10
Silty sandstone	4.8	2500	11.47	0.27	35.08	24.6	3.7	6
No. 8 coal seam	0.6	1400	3.07	0.21	1.02	12.3	1.32	10
Clay mudstone	1.8	2500	21.02	0.25	21.78	15.5	3.21	10
Silty sandstone	8.5	2500	11.47	0.27	35.08	24.6	3.7	6
Limestone	2.7	2700	52.52	0.25	14.23	52.0	3.06	6
Mudstone	0.9	2500	19.96	0.14	32.01	10.3	2.76	10
No. 10 coal seam	0.8	1400	3.07	0.25	1.02	12.3	1.32	10
Fine sandstone	20	2500	20.04	0.28	22.12	15.6	7.96	6

## Results and discussion

### Caving characteristics of overlying strata during coal seam group mining

**Fig. 3:**
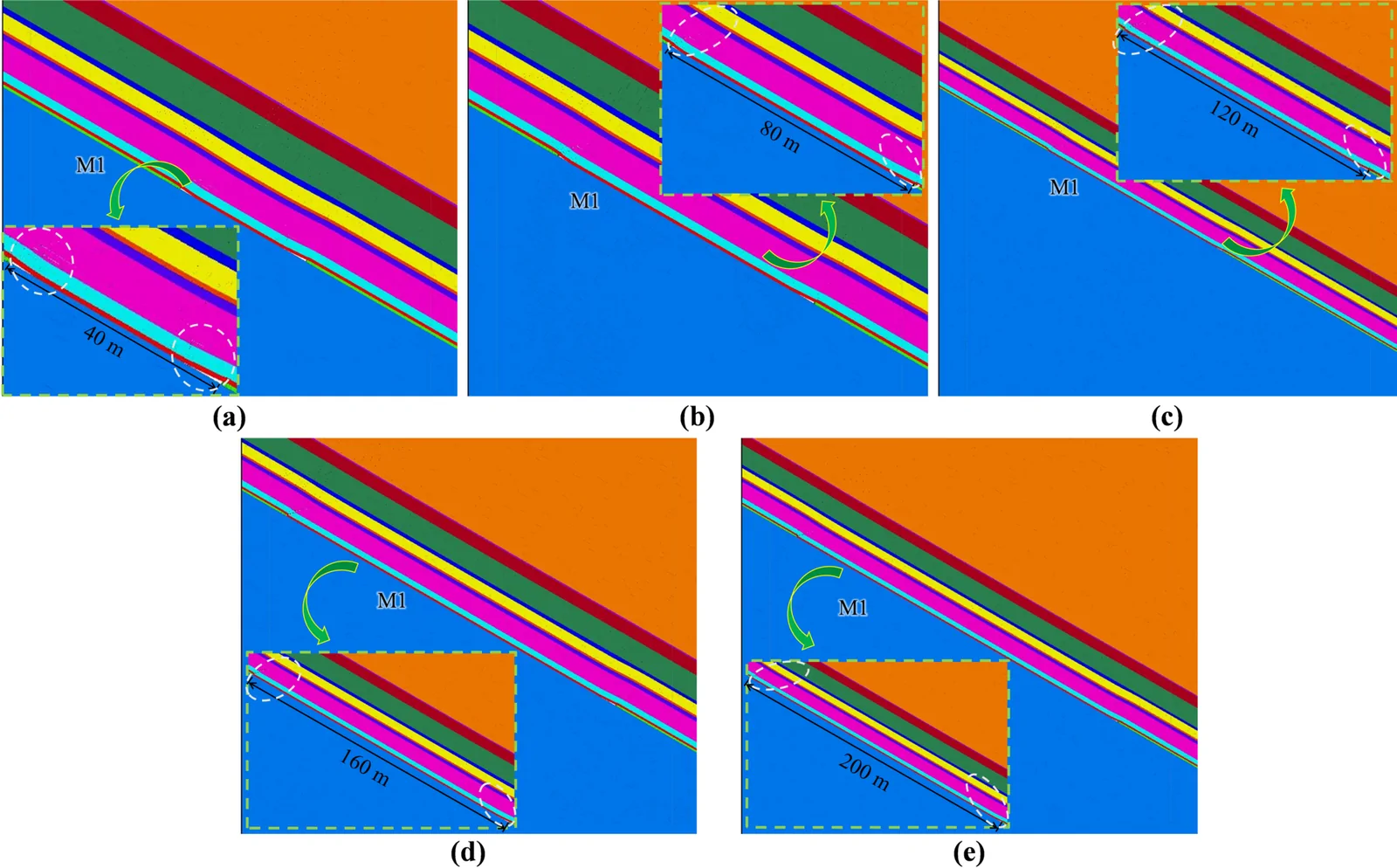
Caving characteristics of overlying strata during M1 mining. (**a**) 40 m; (**b**) 80 m; (**c**) 120 m; (**d**) 160 m; (**e**) 200 m.

**Fig. 4:**
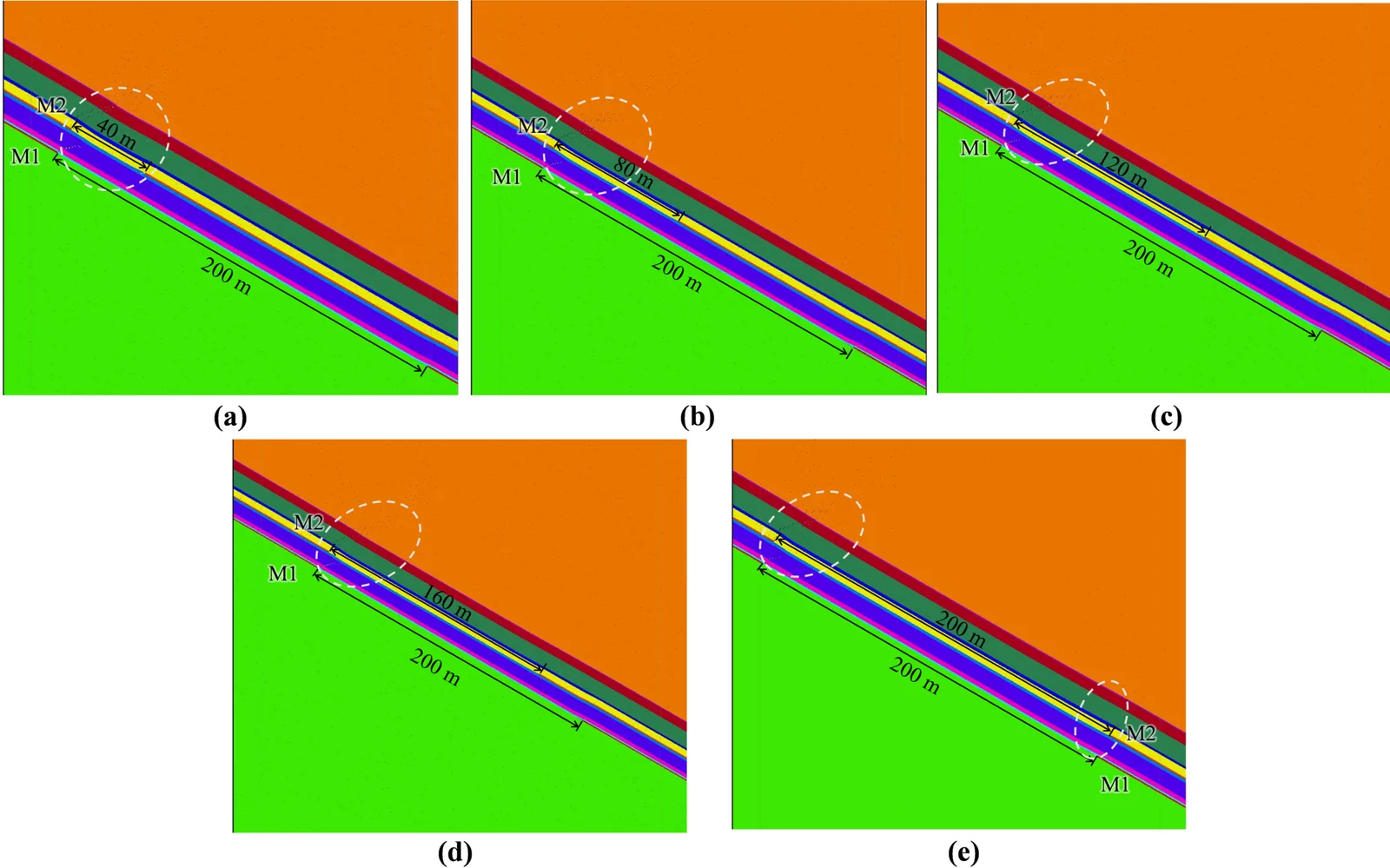
Caving characteristics of overlying strata during M2 mining. (**a**) 40 m; (**b**) 80 m; (**c**) 120 m; (**d**) 160 m; (**e**) 200 m;

**Fig. 5:**
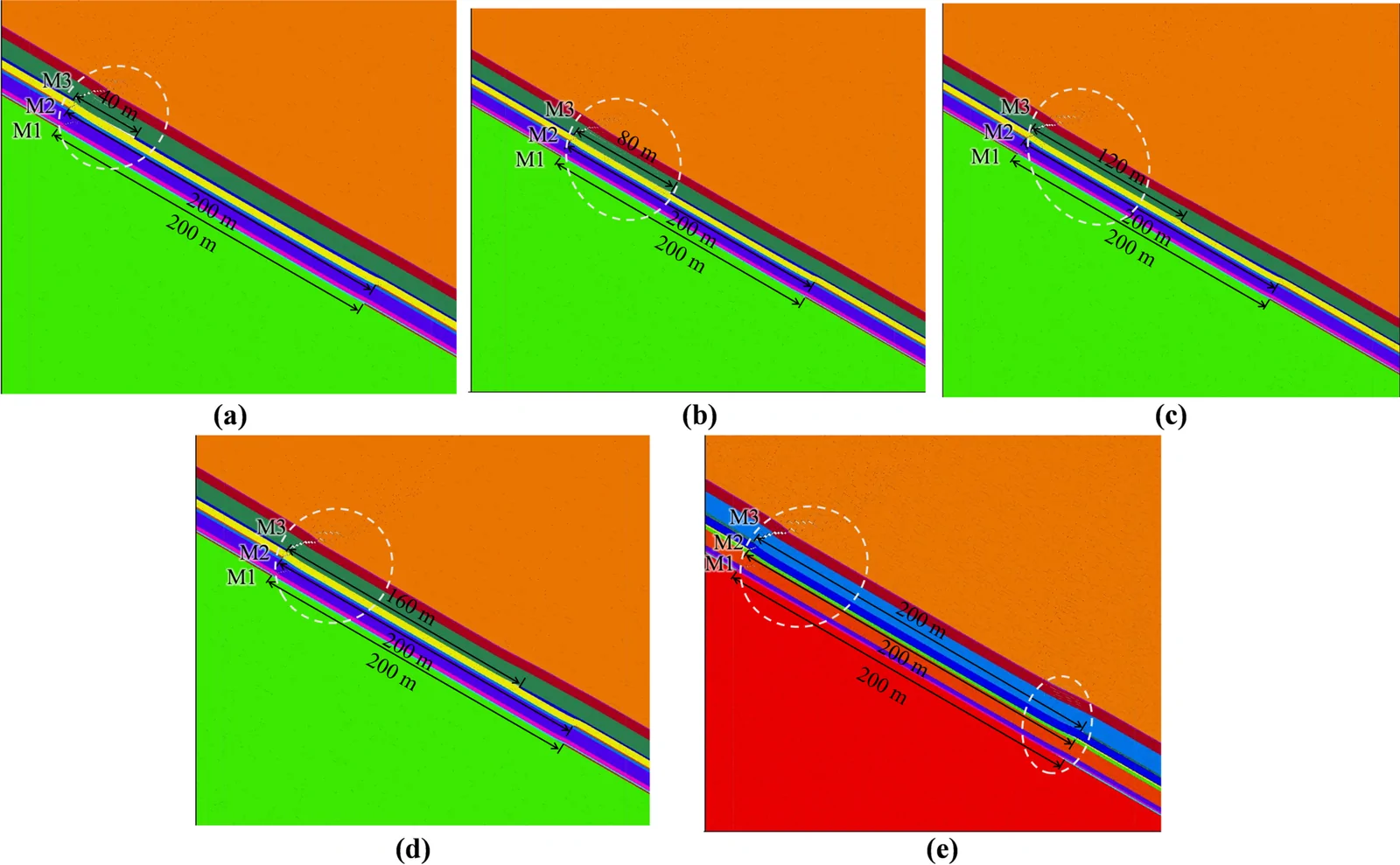
Caving characteristics of overlying strata during M3 mining. (**a**) 40 m; (**b**) 80 m; (**c**) 120 m; (**d**) 160 m; (**e**) 200 m;

Figures 3, 4, and 5 present the structural response characteristics of the overlying strata in the inclined thin coal seam group at advance distances of 40 m, 80 m, 120 m, 160 m, and 200 m during the M1, M2, and M3 mining stages, respectively. Here, M1, M2, and M3 denote the first, second, and third extractions of the three coal seams from bottom to top, respectively. The gray dashed circles indicate the local caving and failure zones. Overall, during repeated mining of the inclined thin coal seam group, the response of the overlying strata exhibits pronounced stagewise, inherited, superimposed, and asymmetric characteristics. As the mined seam progressively shifts upward and the advance distance continues to increase, the deformation and failure range of the overlying strata expands continuously, the height of fracture development gradually migrates upward, and the interlayer disturbance effect becomes increasingly significant.

During the M1 mining stage, because the thickness of the first mined seam was relatively small, the disturbance exerted on the overlying strata was limited, and no obvious intense caving phenomenon was observed overall. As shown in Fig. 3, as the working face advanced from 40 m to 200 m, the overlying strata were characterized mainly by post-mining subsidence deformation and local fracture development, while gradually compacting the underlying goaf during subsidence. In particular, fracture development induced by stratum bending and local instability can be observed on the left side of the goaf; however, no caving structure with a clear boundary and obvious extent was formed. This indicates that, under the first mining of a thin coal seam, the response of the overlying strata was dominated by bending subsidence, fracture initiation, and goaf compaction, with an overall low degree of damage and inconspicuous caving characteristics.

During the M2 mining stage (Fig. 4), because extraction of the middle seam was superimposed on the disturbance generated after M1 mining, the underlying goaf and the associated interlayer weakening effect began to manifest, making the overlying strata more susceptible to deformation and failure during the second extraction. As the advance distance increased from 40 m to 200 m, both the subsidence magnitude and fracture development range of the overlying strata above the M2 panel became more pronounced than those in the M1 stage, the bending deformation of the interlayered strata was further intensified, and local breakage features gradually emerged. Meanwhile, the mining-induced effect of M2 overlapped with the residual disturbed zone left by M1, causing the structural response of the overlying strata to evolve from local deformation during the first extraction to a coordinated instability process under the influence of multi-seam mining.

During the M3 mining stage, extraction of the upper seam was superimposed on the disturbance base formed by the previous two mining stages, and the overlying strata experienced more significant repeated disturbance and structural weakening. As shown in Fig. 5, with continued advance of the working face, subsidence, fracture propagation, and local breakage of the overlying strata were all further intensified, the disturbance interaction among multiple goafs became more evident, and the failure range of the overlying strata continued to extend upward. Compared with the M1 and M2 stages, the overall response of the overlying strata was strongest during the M3 stage, indicating that the cumulative damage effect and interlayer superimposed instability effect of the overlying strata were significantly enhanced after repeated mining.

It should be noted that, under the influence of seam inclination, the caving and fracture development of the overlying strata exhibit a pronounced spatially asymmetric distribution. Because gravity has a component acting along the dip direction, the overlying strata are more prone to sliding, tensile cracking, and bed separation on the upper side along the dip after mining disturbance, resulting in more pronounced caving and failure in the upslope direction. In contrast, the overlying strata on the lower side are characterized mainly by compaction and relatively constrained deformation, with a comparatively smaller failure range. This indicates that, under inclined conditions, stratum sliding and bed separation are more prominent, and the structural response of the overlying strata no longer exhibits the approximately symmetric failure observed under horizontal seam conditions, but instead shows a distinctly non-uniform evolutionary pattern along the dip direction.

In summary, during bottom-up repeated mining of the inclined thin coal seam group, the structural response of the overlying strata was not a simple repetition of single-mining-induced behavior, but a dynamic evolutionary process progressing from weak to strong, from local to overall response, and from localized fracture development to superimposed failure under multi-seam disturbance. During the M1 stage, owing to the small seam thickness, the overlying strata mainly exhibited subsidence compaction and local fracture development, with no obvious caving. During the M2 stage, on the basis of the initial disturbance induced by the first extraction, overlying strata deformation and fracture propagation were further intensified. During the M3 stage, under the superimposed effects of the previous two mining events, both the failure range and structural response intensity of the overlying strata reached their maximum. Meanwhile, controlled by the inclined structure, the caving and fracture evolution of the overlying strata consistently exhibited pronounced dip-direction asymmetry, characterized by stronger failure on the upper side and relatively constrained deformation on the lower side. These findings indicate that, under repeated mining conditions, the response of the overlying strata is governed by both a significant cumulative amplification effect and a strong inclination-control effect, thereby providing a basis for the subsequent analysis of stress redistribution, fracture propagation, and hazard evolution mechanisms under repeated mining disturbance.

### Stress distribution characteristics of the overlying strata during coal seam group mining

#### Characteristics of vertical stress contours

**Fig. 6:**
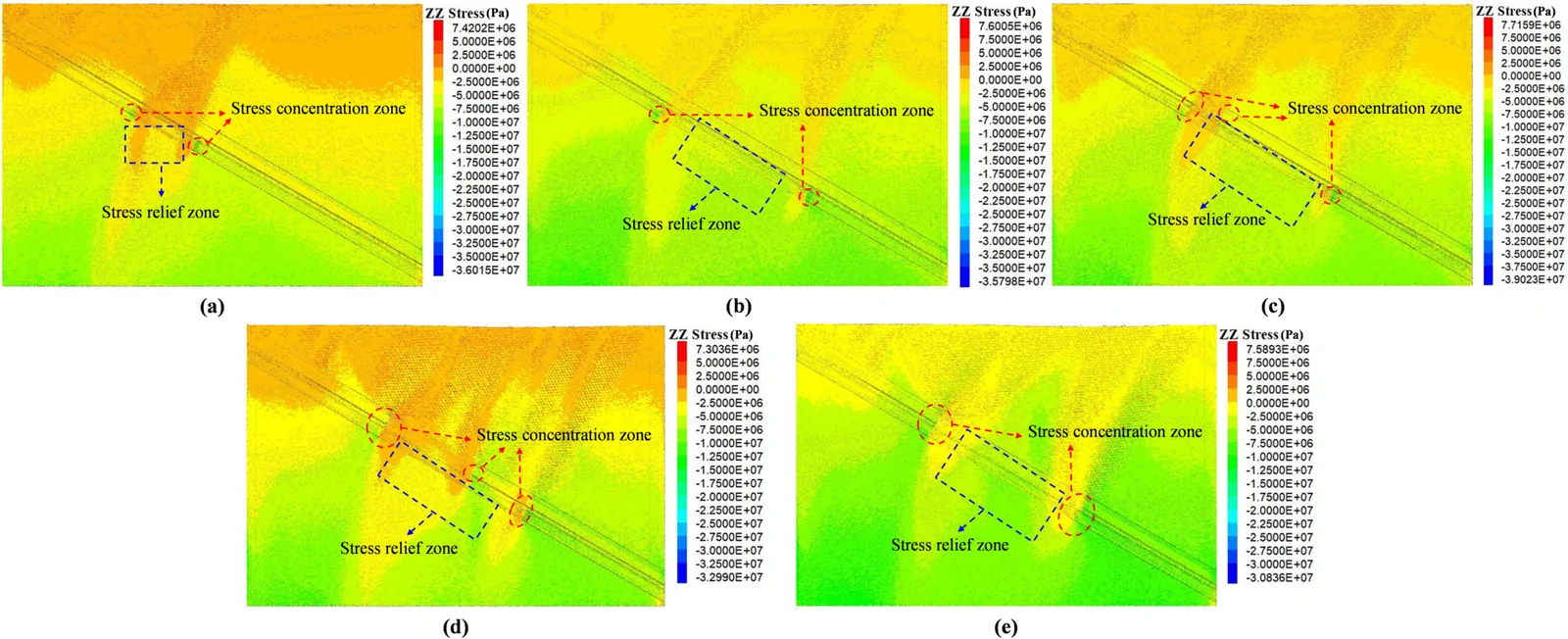
Vertical stress contours of the overlying coal-rock mass during mining of the inclined thin coal seam group. (**a**) First seam mined to 80 m; (**b**) first seam mined to 200 m; (**c**) second seam mined to 40 m; (**d**) third seam mined to 120 m; (**e**) third seam mined to 200 m.

As shown in Fig. 6, during mining of the inclined thin coal seam group, the vertical stress field in the overlying strata exhibits pronounced characteristics of dynamic redistribution, stagewise evolution, and dip-direction asymmetric response. As the mined seam progressively shifts upward and the working face advance increases, the original stress equilibrium is continuously disturbed. A distinct stress-relief zone gradually develops within and above the goaf, whereas persistent stress concentration zones form ahead of the working face and near the goaf boundaries.

As shown in Fig. 6a, when the first seam was mined to 80 m, the range of mining-induced disturbance was relatively limited, and stress redistribution was concentrated mainly in the vicinity of the working face. Local stress relief had already occurred within and above the goaf, while initial stress concentration zones formed ahead of the working face and near the goaf boundaries. As shown in Fig. 6b, as the first seam advanced to 200 m, the goaf expanded significantly, and subsidence and structural adjustment of the overlying strata became more pronounced. The stress-relief zone continued to expand, whereas the stress concentration zone migrated forward with the advancing working face and became increasingly intensified, indicating that the load-bearing structure of the overlying strata had undergone substantial adjustment under first-mining conditions. As shown in Fig. 6c, upon entering the 40 m advance stage of the second seam, the stress field in the overlying coal-rock mass no longer exhibited the initial response characteristic of single-seam mining, but instead showed a pronounced superimposed evolutionary pattern due to the influence of the underlying disturbance caused by the first extraction. The stress-relief effect left by mining of the first seam still persisted, while mining of the second seam induced new stress concentration zones in adjacent areas, resulting in the coexistence of goaf stress relief and concentrated loading ahead of the working face, thereby markedly increasing the complexity of the stress field. As shown in Figs. 5e and 6d, when mining advanced to 120 m and 200 m in the third seam, the superimposed effect of multi-seam mining was further strengthened, and the overlying coal-rock mass experienced continuous repeated disturbance, leading to more pronounced stress differentiation. At this stage, the stress-relief zone above the multiple goafs continued to expand, whereas the stress concentration zones ahead of the working face and near the goaf boundaries further developed and migrated forward, reflecting continuous reconstruction of the load-bearing structure of the overlying strata under repeated mining, together with a gradual enlargement of the overall instability range.

In addition, under the control of seam inclination, the vertical stress field exhibits a marked asymmetric distribution along the dip direction. Because gravity has a component acting along the dip direction, the overlying strata are more prone to non-uniform subsidence, interlayer sliding, and separation-induced tensile cracking after mining, resulting in significant differences in stress responses between the high side and the low side. Overall, the upper side along the dip direction, namely the high side, shows a larger range of rock mass failure and stress relief, whereas the lower side along the dip direction, namely the low side, is characterized by more pronounced compaction. This indicates that the evolution of mining-induced stress under inclined conditions is strongly governed by structural control.

#### Evolution of vertical stress curves

**Fig. 7:**
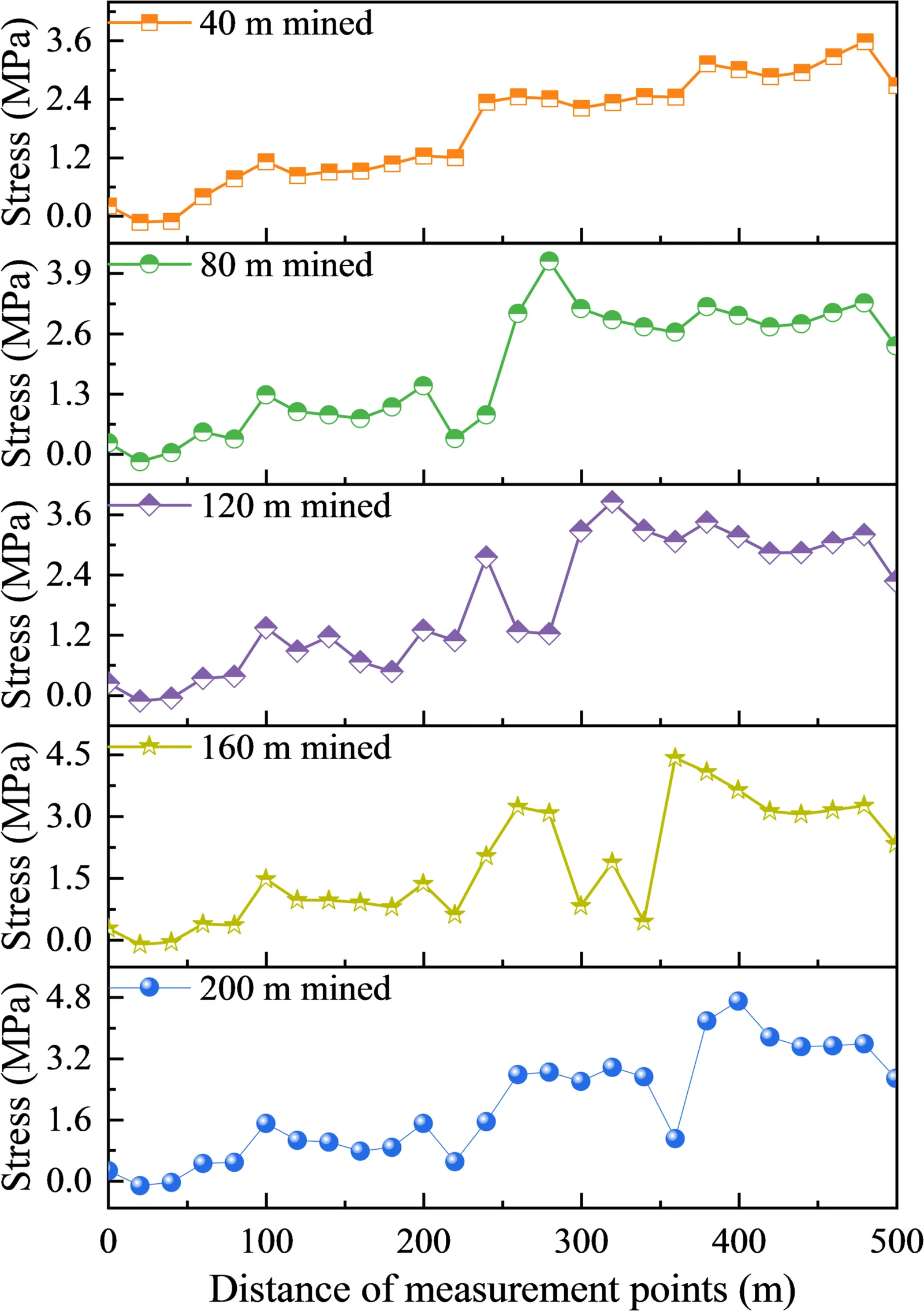
Stress evolution of the overlying coal-rock mass during M1 mining.

**Fig. 8:**
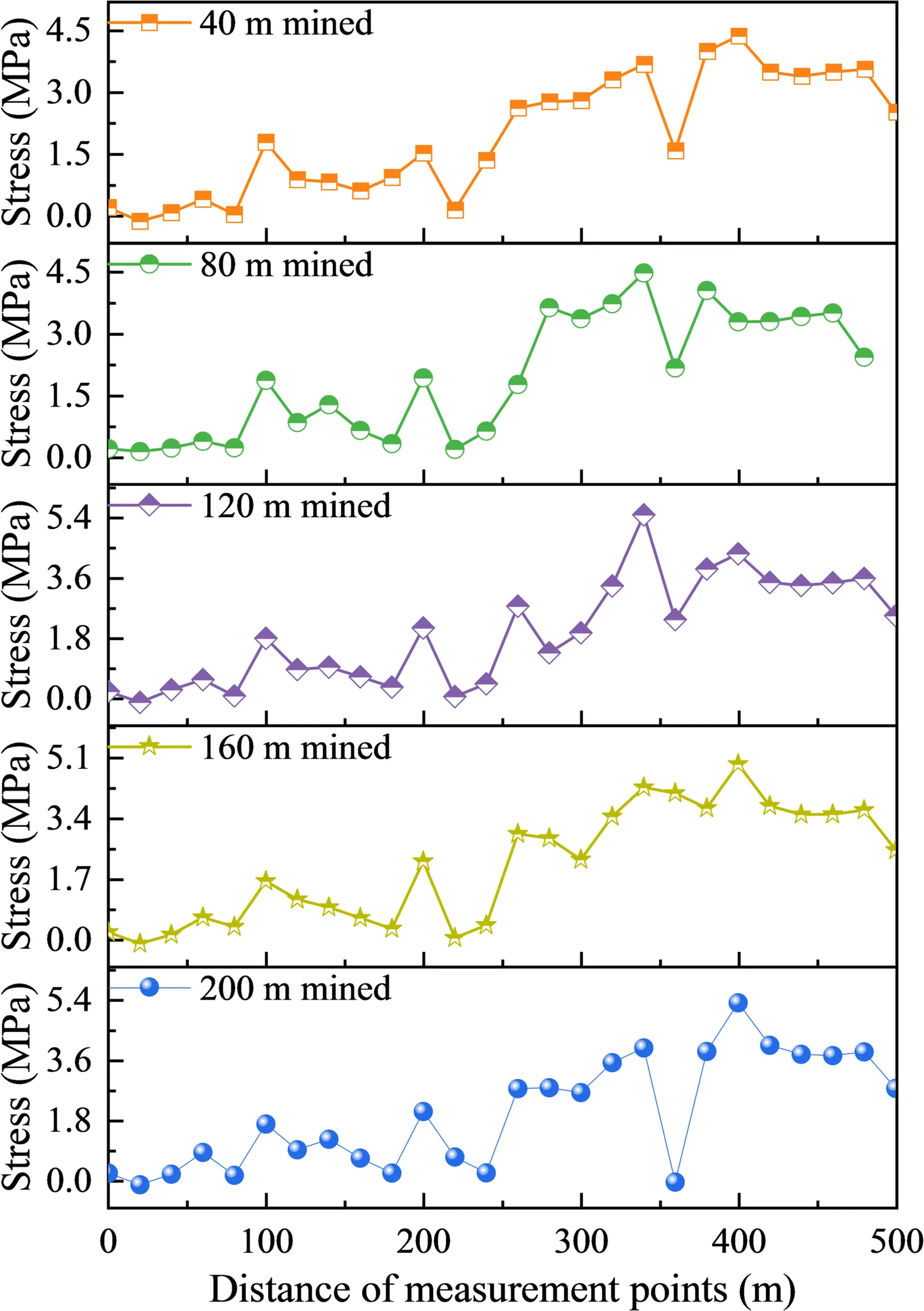
Stress evolution of the overlying coal-rock mass during M2 mining.

**Fig. 9:**
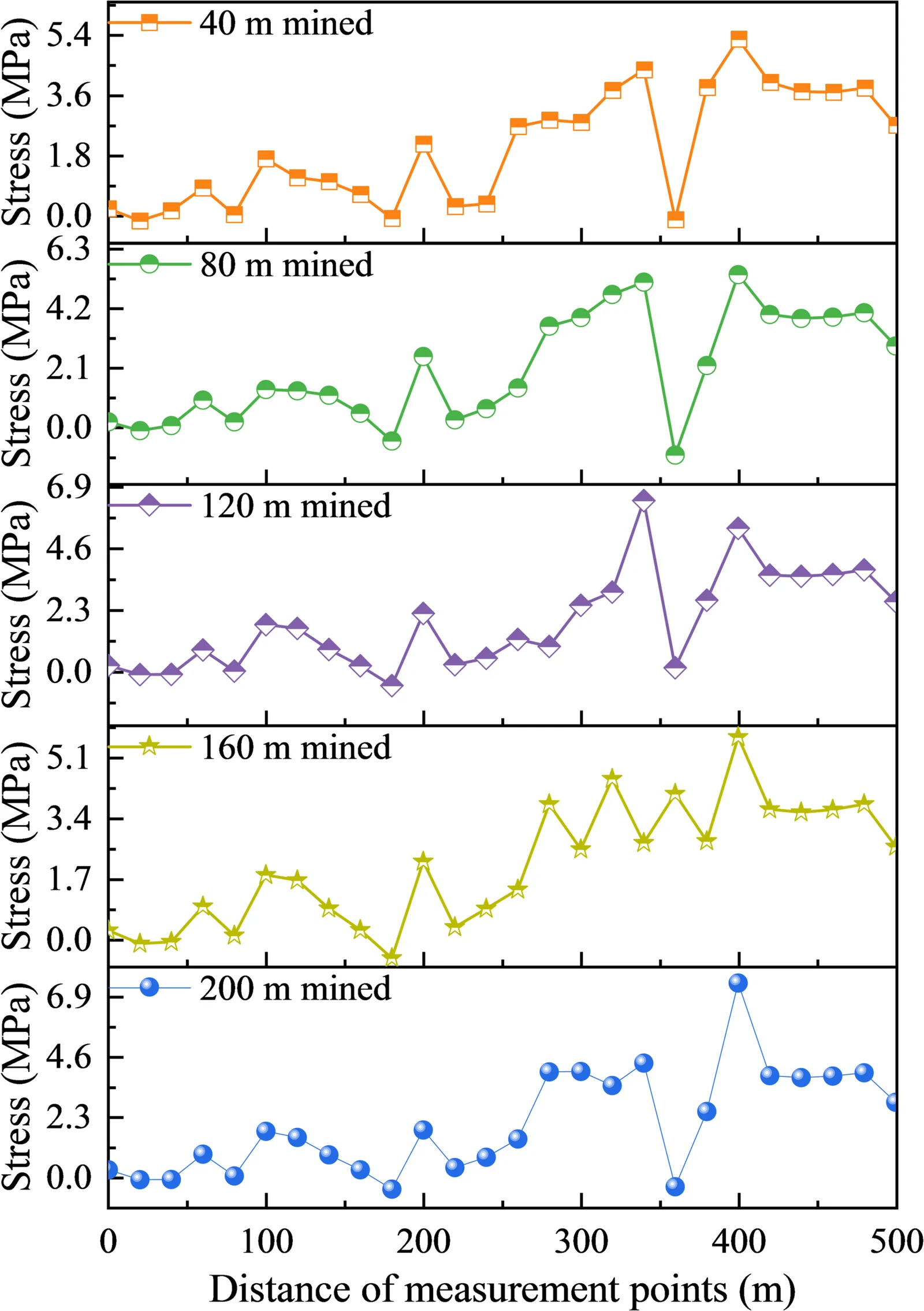
Stress evolution of the overlying coal-rock mass during M3 mining.

As shown in Figs. 7, 8 and 9, during repeated mining of the inclined thin coal seam group, the stress evolution recorded at monitoring points located at different positions along the dip direction of the No. 6 coal seam exhibits pronounced zonal, stagewise, and asymmetric characteristics. In general, before being strongly affected by mining disturbance, the stress at each monitoring point varies gradually along the dip direction, and the overall curve remains consistent with the original stress distribution under inclined seam occurrence conditions, with only minor fluctuations. However, as the mined seam shifts upward and the mining range expands, the stress field gradually evolves from an initially gentle distribution into a non-uniform pattern characterized by stress concentration on both sides and stress relief in the middle.

During the M1 mining stage (Fig. 7), the overall stress variation at each monitoring point is relatively gentle, and obvious disturbance occurs only in local areas. After the working face advanced, the stress at monitoring points located approximately 100 m and 380 m along the dip direction increased significantly, forming local stress peaks corresponding to the concentrated loading zones on both sides of the goaf. In contrast, the stress at monitoring points between these two peaks decreased overall, exhibiting a distinct stress-relief characteristic. This indicates that an initial stress redistribution had already occurred in the overlying coal-rock mass under first-mining conditions, although the affected range and disturbance intensity remained relatively limited. Upon entering the M2 mining stage (Fig. 8), owing to the residual stress relief and structural weakening left by M1 mining, the amplitude of stress fluctuations at each monitoring point increased markedly, and the stress differentiation along the dip direction became more pronounced. On the one hand, the original stress-relief zone still persisted; on the other hand, the second mining event induced new local stress concentration peaks, making the stress responses among different monitoring points more distinct. This indicates that the stress field had evolved from local adjustment during the first mining stage to a superimposed evolutionary process under repeated mining disturbance. During the M3 mining stage (Fig. 9), the superimposed effect of multi-seam mining was further intensified, the stress curves showed the strongest fluctuations, and local peak-trough variations became more evident. Compared with the M1 and M2 stages, the magnitude of the concentrated stress zones increased further, whereas some monitoring points that had previously borne load entered a clearly stress-relieved state. In particular, the stress at the monitoring point near 380 m decreased, indicating a further expansion of the stress-relief zone. This demonstrates that, with continued advance of the third seam, the load-bearing structure of the overlying coal-rock mass was continuously reconstructed, and mining-induced disturbance evolved from local stress adjustment into broader stress transfer and redistribution.

In addition, controlled by the seam dip angle, the stress evolution at monitoring points along the dip direction consistently exhibits a pronounced asymmetric character. Under inclined conditions, the gravitational component acting along the dip direction alters the load transfer path and structural response mode of the overlying strata, resulting in significant differences in the degree of stress concentration, magnitude of stress relief, and evolutionary process between the high side and the low side. Overall, mining of the first seam generated the initial zonation of stress concentration and stress relief, whereas mining of the second and third seams progressively intensified the stress superposition effect and expanded the stress-relief range on this basis, ultimately forming an asymmetric stress evolution pattern controlled by the inclined structure. These results provide a mechanical basis for further elucidating the deformation and failure of the overlying strata, fracture evolution, and hazard development mechanisms during mining of the inclined thin coal seam group.

The stress redistribution described above directly controls the subsequent displacement and fracture evolution of the overlying strata. Stress relief within the goaf reduces the local bearing capacity and promotes roof subsidence, whereas stress concentration near the working face and goaf boundaries enhances load transfer and local deformation. Under inclined conditions, the asymmetric distribution of stress relief and stress concentration causes differential movement between the high side and low side, leading to uncoordinated subsidence, interlayer sliding, and further tensile–shear fracture development. Thus, the displacement evolution analyzed in the following section is the deformation response to mining-induced stress redistribution, and fracture development is the result of continued structural weakening under differential subsidence.

### Displacement distribution characteristics of the overlying strata during coal seam group mining

#### Characteristics of vertical displacement contours

**Fig. 10:**
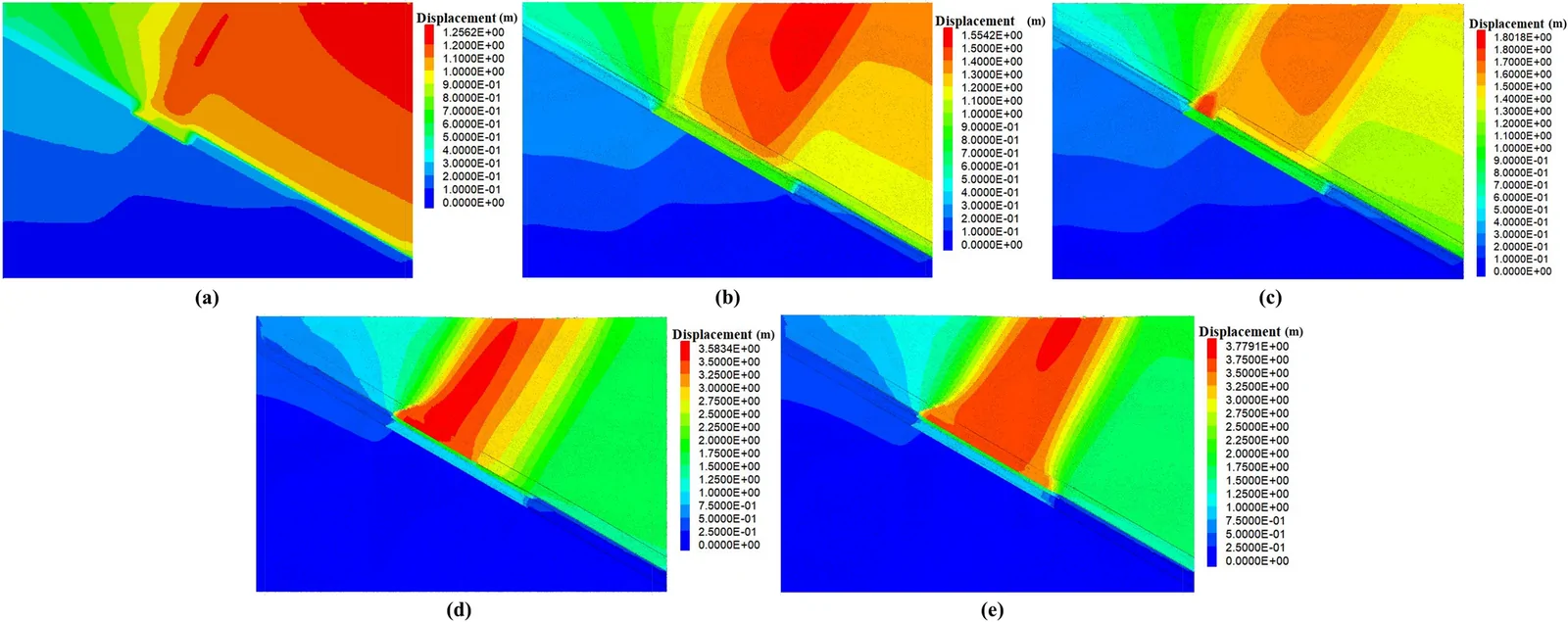
Displacement contours of the overlying strata during mining of the inclined coal seam group. (**a**) First seam mined to 80 m; (**b**) first seam mined to 200 m; (**c**) second seam mined to 40 m; (**d**) third seam mined to 120 m; (**e**) third seam mined to 200 m.

As shown in Fig. 10, during mining of the inclined coal seam group, the displacement field of the overlying coal-rock mass exhibits pronounced cumulative expansion and dip-direction asymmetric response as the mined seam progressively shifts upward and the advance distance increases. In general, mining disturbance disrupts the initial mechanical equilibrium, causing the displacement above the goaf and in adjacent areas to increase continuously, while the affected displacement zone extends progressively upward into the deeper overlying strata and laterally along both sides of the dip direction. Meanwhile, under the control of seam inclination, the displacement contours display a distinctly inclined distribution, indicating that the movement of the overlying strata is strongly non-uniform.

As shown in Fig. 10a, when the first seam was mined to 80 m, the goaf remained relatively small, and only local subsidence and bending deformation occurred in the overlying strata near the working face and above the goaf. The overall displacement was limited, and the displacement contours gradually attenuated from the goaf toward the outer regions, indicating that overlying strata movement during the first mining stage was mainly confined to a local area. As shown in Fig. 10b, as the first seam advanced to 200 m, the goaf expanded significantly, and the bending subsidence of the overlying strata became markedly more pronounced, with the displacement-affected zone extending further upward, indicating that mining of the first seam had already caused a continuous increase in the overall deformation range of the overlying strata. As shown in Fig. 10c, upon entering the 40 m advance stage of the second seam, further displacement adjustment occurred in the overlying coal-rock mass on the basis of the deformation induced by mining of the first seam. Although the advance distance of the second seam was relatively short, a new zone of increased displacement had already formed near the new working face, indicating that the secondary disturbance induced by repeated mining had begun, and that the displacement field of the overlying strata was gradually evolving from local development under first-mining conditions to a superimposed evolutionary process under multi-seam mining. As shown in Fig. 10d, when mining of the third seam advanced to 120 m, the goafs formed by extraction of the first two seams had already caused substantial disturbance to the overlying strata, and the displacement range expanded further. The high-displacement zone shifted markedly upward and extended along the dip direction, indicating that the overall deformation effect of the overlying strata continued to intensify under repeated mining conditions. As shown in Fig. 10e, as the third seam advanced to 200 m, the superimposed effect of multiple goafs reached its strongest level, and a larger continuous high-displacement zone developed in the overlying coal-rock mass. The overall response of the overlying strata was dominated by subsidence deformation, indicating that its load-bearing structure had undergone significant reconstruction and that the mining-induced effect had evolved from local deformation into an integral subsidence response.

In addition, owing to the effect of seam inclination, the displacement field exhibits a pronounced asymmetric distribution along the dip direction. Because gravity has a component acting along the dip direction, the overlying strata are more prone to sliding, bed separation, and subsidence propagation toward the high side of the dip after mining. Consequently, the high-displacement zone and displacement contours shift markedly toward one side, whereas the opposite side is characterized mainly by relatively stronger compaction and constrained deformation. This phenomenon indicates that, under inclined conditions, the displacement evolution of the overlying strata does not follow the symmetric subsidence mode typical of horizontal seams, but rather represents a non-uniform deformation process controlled by seam dip.

Overall, during repeated mining of the inclined coal seam group, the displacement field of the overlying coal–rock mass evolves from local subsidence in the first mining stage to superimposed expansion under repeated mining disturbance, and finally to overall asymmetric subsidence under multi-seam mining conditions. Its main characteristics can be summarized as follows: displacement above the goaf continuously increases; the high-displacement zone progressively migrates upward and expands; the superimposed effect of multi-seam mining significantly intensifies the overall deformation of the overlying strata; and, under the control of the inclined structure, a pronounced asymmetric displacement distribution pattern is ultimately formed.

#### Evolution of vertical displacement curves

**Fig. 11:**
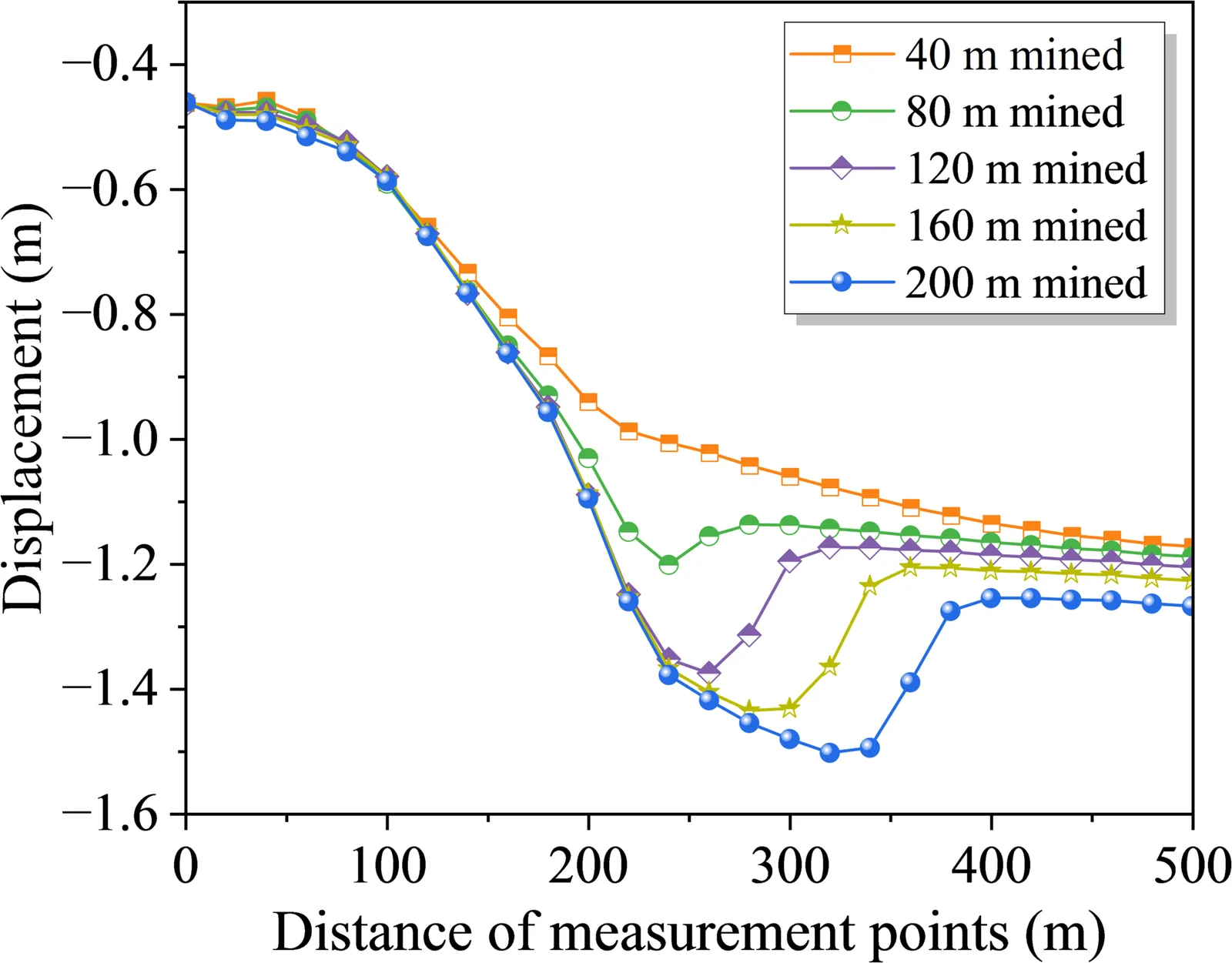
Evolution of vertical displacement of the overlying strata during M1 mining.

**Fig. 12:**
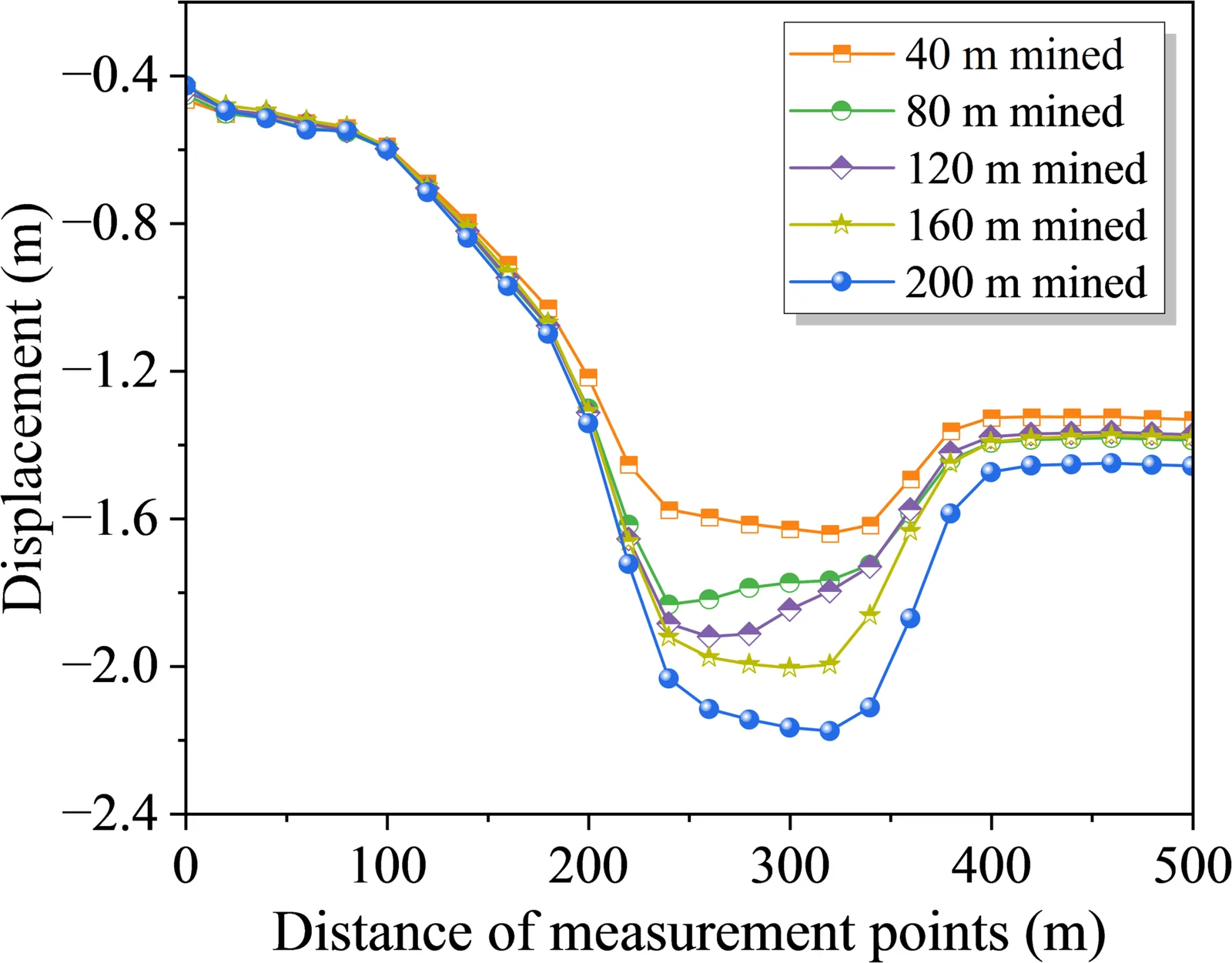
Evolution of vertical displacement of the overlying strata during M2 mining.

**Fig. 13:**
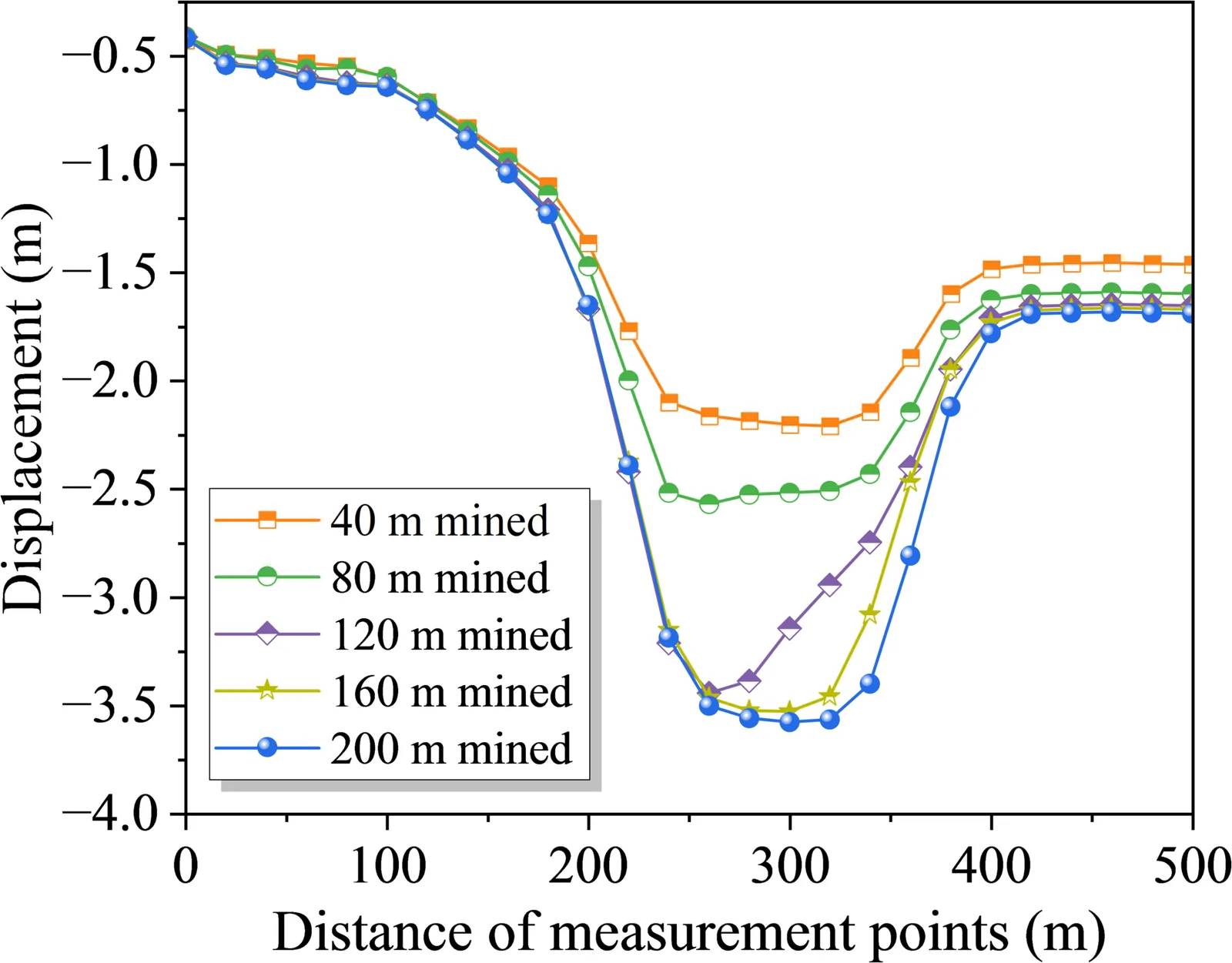
Evolution of vertical displacement of the overlying strata during M3 mining.

As shown in Figs. 11, 12 and 13, during repeated mining of the inclined coal seam group, the vertical displacement evolution of the overlying strata along the dip direction exhibits pronounced stagewise, cumulative, and asymmetric characteristics. In general, as the working face advances from 40 to 200 m, the subsidence troughs of the vertical displacement curves along each monitoring line continuously deepen and widen, indicating that goaf expansion progressively increases both the subsidence range and magnitude of the overlying strata. Meanwhile, under the control of seam inclination, the displacement curves generally exhibit a distinctly asymmetric concave profile, suggesting that subsidence of the overlying strata under inclined conditions does not develop symmetrically.

During the M1 mining stage (Fig. 11), as the advance distance increased, the lowest point of the vertical displacement curve continued to move downward, and the subsidence trough gradually widened, indicating that noticeable bending subsidence and displacement redistribution had already occurred in the overlying strata under first-mining conditions. Overall, the variation in the subsidence curves during the M1 stage remained relatively moderate, indicating that the disturbance was concentrated mainly above the goaf and in adjacent areas, and that movement of the overlying strata was still dominated by local deformation. Upon entering the M2 mining stage (Fig. 12), because mining of the first seam had already caused preliminary disturbance and structural weakening in the overlying strata, the vertical displacement induced by extraction of the second seam increased significantly, the lowest point of the curve dropped further, and the asymmetric concave characteristic became more pronounced. This indicates that, under repeated mining conditions, overlying strata subsidence was not a simple continuation of the deformation induced by the first mining stage, but rather a further superposition and amplification based on the earlier extraction, with the cumulative effect of multi-seam mining beginning to emerge. During the M3 mining stage (Fig. 13), the goafs and fracture development resulting from extraction of the first two seams had already substantially weakened the overall stability of the overlying strata, causing the vertical displacement to increase further after mining of the third seam and producing deeper and wider subsidence troughs. Compared with the M1 and M2 stages, the M3 stage exhibited the greatest subsidence magnitude and the broadest influence range of the overlying strata, indicating that the structure of the overlying strata underwent continued instability under repeated mining, with integral subsidence becoming more pronounced.

In addition, controlled by seam inclination, the vertical displacement curves in all three stages exhibit pronounced lateral asymmetry. Under inclined conditions, the overlying strata are more prone to subsidence and sliding toward the low side under the action of the gravitational component along the dip direction, resulting in a greater subsidence magnitude on the low side and relatively weaker deformation on the high side. Therefore, under inclined coal seam group mining conditions, the evolution of vertical displacement in the overlying strata is governed not only by the advance distance and mining horizon but also strongly constrained by the inclined structural setting.

Overall, during repeated mining of the inclined coal seam group, the vertical displacement of the overlying strata evolves from local subsidence during the first mining stage to enhanced subsidence under repeated mining superposition, and finally to overall asymmetric subsidence under multi-seam disturbance. Its basic pattern can be summarized as follows: as the mined seam shifts upward and the advance distance increases, the subsidence trough continuously deepens and widens, the cumulative amplification effect of repeated mining disturbance steadily intensifies, and an asymmetric subsidence mode controlled by the inclined structure is ultimately formed.

### Fractal analysis of failure characteristics during coal seam group mining

It should be noted that the fracture images obtained from the 3DEC numerical simulation are affected by the block discretization scheme, contact topology, and mesh division. The fractures shown in the numerical results mainly represent tensile or shear failure between adjacent blocks, rather than natural fractures in a strict geological sense. Therefore, the fractal dimension calculated in this study should not be interpreted as an absolute quantitative characterization of natural fracture networks. Instead, under the same model discretization, image-processing procedure, and box-counting method, it is used as an auxiliary and relative indicator to describe the complexity variation of numerically simulated fracture traces during different mining stages. Accordingly, the following fractal analysis focuses on the relative evolution trend of simulated fracture complexity from M1 to M3, rather than on the absolute fractal properties of real rock fractures.

To quantitatively characterize the complexity and evolutionary features of the fracture network in the overlying strata during mining of the inclined thin coal seam group, fractal dimension was introduced to analyze fracture morphology^56,57^. Fractal dimension can reflect the space-filling capacity of fracture geometry and its scale self-similarity, and is therefore an important parameter for evaluating the degree of fracture network development, complexity, and connectivity potential. For two-dimensional fracture images, the fractal dimension generally ranges between 1 and 2. A smaller fractal dimension indicates a relatively simple fracture distribution and a lower degree of development, whereas a larger fractal dimension indicates increases in fracture number, branching degree, and spatial propagation capacity, implying that the fracture network tends to become more complex and interconnected.

**Fig. 14:**
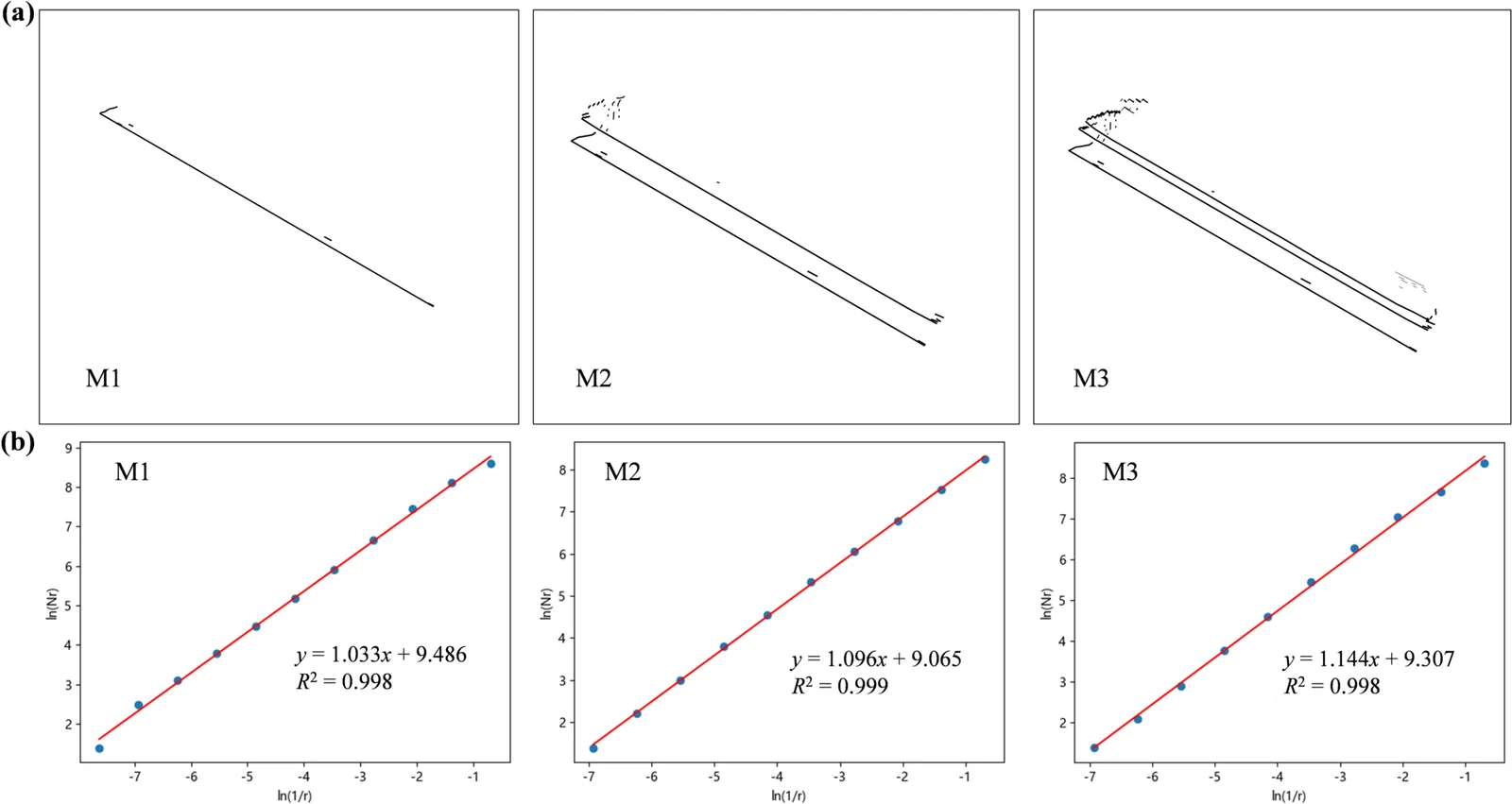
Fractal calculation process of fractures during mining of the inclined thin coal seam group. (**a**) Binary image; (**b**) fractal calculation results.

In this study, the box-counting method was adopted to calculate the fractal dimension of fractures in the overlying strata^58–60^. First, the fracture images obtained at different mining stages were converted into grayscale and then binarized to extract fracture boundary information, thereby generating binary images for fractal analysis, as shown in Fig. 14a. Subsequently, square grids with side length *r* were superimposed on the binary images, and the number of grids containing fracture pixels, *N*(*r*), was counted. If the fracture network possesses fractal characteristics, *N*(*r*) and r satisfy the following power-law relationship:

1 $$N(r) \propto {r^{ - D}}$$

where *N*(*r*) is the number of grids with side length *r* required to cover the fracture target, and *D* is the fractal dimension of the fracture network. Taking the natural logarithm of both sides of Eq. (1) yields:

2 $$\ln N(r)=D\ln (1/r)+C$$

where *C* is a constant. Therefore, by performing linear fitting with *łn (1/r)* as the abscissa and *łn N(r)* as the ordinate, the slope of the fitted line gives the fracture fractal dimension *D*. Figure 14b presents the fitting results for the fracture images at different mining stages.

As shown in Fig. 14, the fractal dimensions of fractures at the M1, M2, and M3 stages are 1.023, 1.096, and 1.144, respectively, with corresponding coefficients of determination of *R*^2^ = 0.998, 0.999, and 0.998. These results indicate that the fracture images at all stages exhibit a good fractal scaling relationship, demonstrating that the box-counting method provides a reliable characterization of fracture evolution in the overlying strata during mining of the inclined thin coal seam group. Meanwhile, the binary images show intuitively that, as the mined seam progressively shifts upward from M1 to M3, the fractures evolve from local discrete development toward multi-fracture propagation and interconnection, with a marked increase in the spatial complexity of the fracture network.

**Fig. 15:**
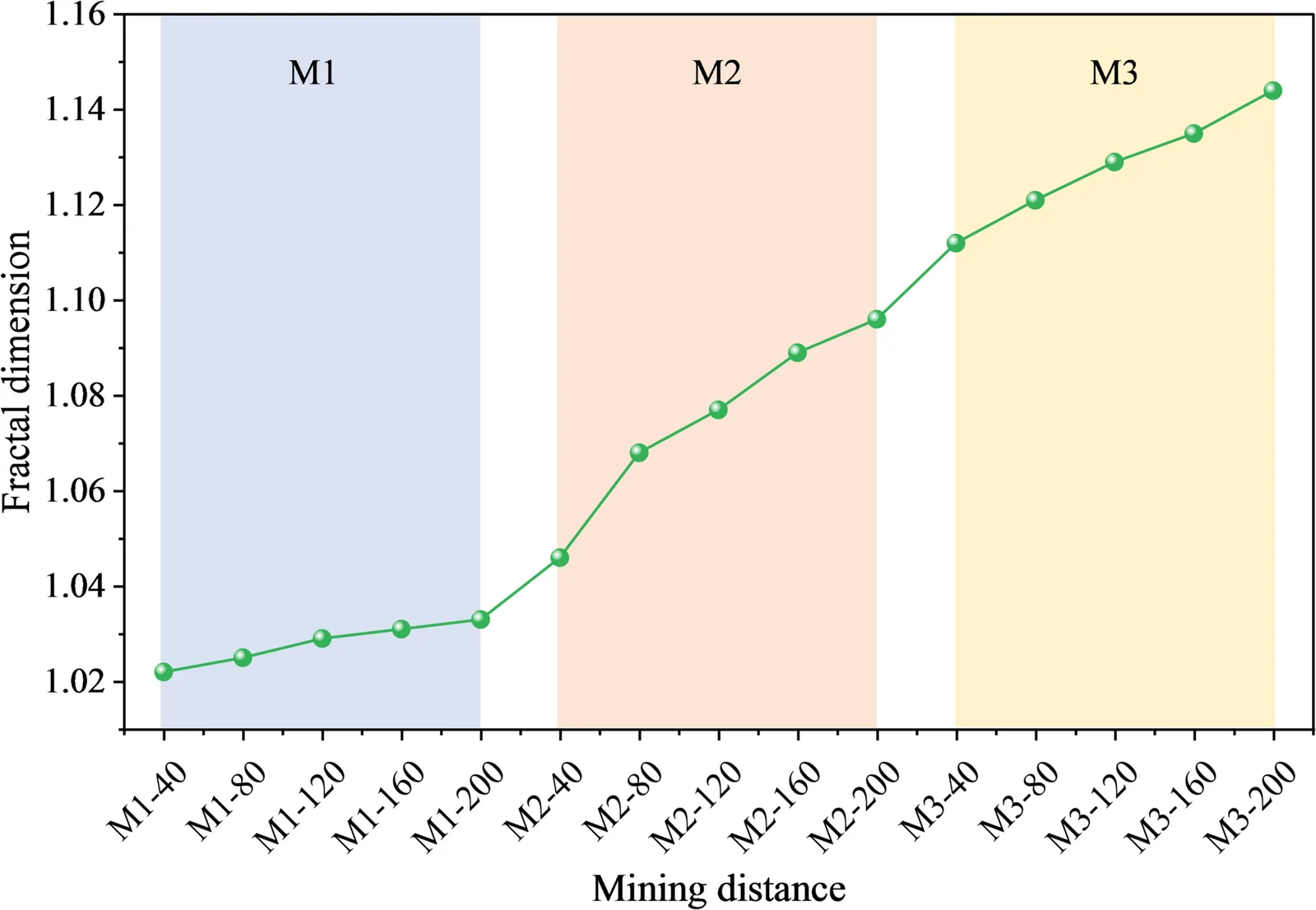
Fractal evolution characteristics of fractures in the overlying strata during mining of the inclined thin coal seam group.

As shown in Fig. 15, the fractal dimension of fractures in the overlying strata exhibits an overall increasing trend with repeated mining, indicating that the complexity, heterogeneity, and space-filling capacity of the fracture system continuously increase. During the M1 stage, the fractal dimension increases only slightly, suggesting that fractures in the overlying strata are still dominated by local initiation and limited propagation under first-mining conditions, and that the overall degree of damage remains relatively low. Upon entering the M2 stage, the fractal dimension increases markedly, indicating that, on the basis of the disturbance induced by the first mining, pre-existing fractures reopen and propagate, while new fractures are continuously generated. Consequently, the fracture network evolves rapidly from a discrete state to a connected state. By the M3 stage, the fractal dimension further increases and reaches its maximum value, indicating that, under repeated multi-seam mining disturbance, fractures in the overlying strata have evolved from local damage into a large-scale, multilevel complex fracture system.

Mechanistically, the continuous increase in fracture fractal dimension reflects the structural evolution of the overlying strata from a relatively intact state to progressive fragmentation, and from local damage to overall structural reconstruction. During the first mining stage, the disturbance induced by thin-seam extraction is limited, and fracture development is controlled mainly by bending subsidence and local tensile deformation. During the second and third mining stages, however, owing to the residual stress-relief effect and structural weakening caused by the earlier goafs, the overlying strata become more susceptible to interlayer separation, sliding, and breakage, resulting in simultaneous increases in fracture number, scale, and connectivity. In particular, under inclined conditions, the gravitational component acting along the dip direction further promotes fracture propagation and structural instability, causing the fracture network to exhibit stronger spatial non-uniformity and greater evolutionary complexity.

Overall, during repeated mining of the inclined thin coal seam group, the fracture fractal dimension of the overlying strata increases from 1.023 to 1.144, indicating that the fracture network undergoes an evolutionary process from local initiation to propagation and multiplication, and finally to complex interconnection. The increase in fractal dimension not only reveals the continuous enhancement of the damage degree and fracture complexity of the overlying strata but also indicates that repeated mining significantly improves the spatial connectivity of the fracture network. This provides a quantitative basis for the subsequent analysis of dominant fracture channel formation and seepage capacity evolution in the overlying strata.

This increasing trend should be understood as a relative increase in the complexity of simulated fracture traces under the same discretization conditions, rather than as a direct measurement of the absolute complexity of natural fracture networks.

### Asymmetric instability-induced hazard-causing mechanism of overlying strata during mining of the inclined thin coal seam group

By integrating the caving characteristics, stress evolution, displacement response, and fracture fractal features of the overlying strata, it can be seen that, under bottom-up repeated mining of an inclined thin coal seam group, the evolution of the overlying strata is not a simple repetition of disturbance induced by a single mined seam, but a progressive instability process jointly controlled by repeated mining effects and inclined structural effects. During the first mining stage, because the seam thickness is relatively small, the response of the overlying strata is manifested mainly as local subsidence, goaf compaction, and limited fracture initiation, while the overall damage remains relatively slight. However, as the overlying seams are successively extracted, the pressure-relief effect, structural weakening effect, and interlayer disturbance inherited from the earlier goafs continue to superimpose, causing the overlying strata to evolve from local deformation to overall asymmetric instability under the coupled control of multi-seam interaction. This indicates that asymmetric instability of the overlying strata is the most representative hazard-causing form during mining of the inclined thin coal seam group, and that its essence lies in the continuous reconstruction of the load-bearing structure and the cumulative development of local instability under repeated mining conditions.

**Fig. 16:**
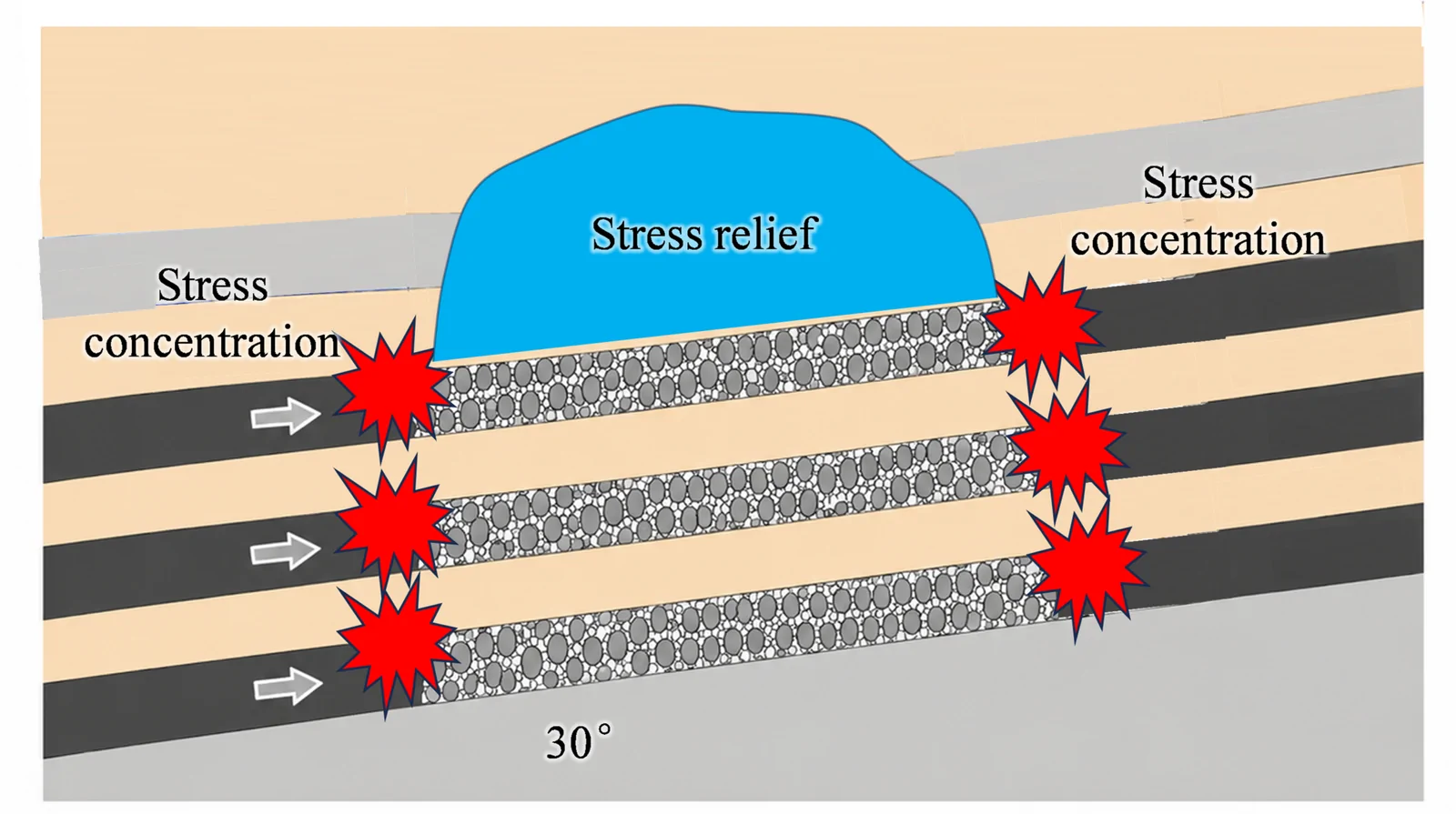
Schematic diagram of stress variation during repeated mining of an inclined thin coal seam group.

From the perspective of stress evolution (Fig. 16), mining first disrupts the original stress equilibrium of the overlying strata, causing the vertical stress field to evolve gradually from an initially gentle distribution into a basic pattern characterized by stress relief within the goaf and concentrated loading ahead of the working face and near the goaf boundaries. As the mined seam shifts upward and the advance distance increases, the concentrated stress zones continue to migrate forward and become locally intensified, while the pressure-relief zones expand continuously, indicating that the load transfer path and load-bearing framework within the overlying strata remain under continuous adjustment. Under the control of seam inclination, the stress field exhibits pronounced differentiation along the dip direction, and clear differences arise between the high side and the low side in terms of concentrated loading, pressure-relief extent, and evolutionary degree, reflecting the marked asymmetry of the stress state of the overlying strata under inclined conditions. Such non-uniform stress redistribution continuously increases the load-bearing demand in local regions and drives them toward the limit state, thereby creating the stress conditions for key stratum breakage, roof instability, and local dynamic manifestations.

From the perspective of deformation evolution, both the displacement field and the vertical displacement curves indicate that, with repeated mining, the subsidence trough continuously deepens and widens, and the high-displacement zone gradually migrates upward and expands toward both sides along the dip direction. The overlying strata thus evolve from local bending subsidence during first mining to overall asymmetric subsidence under multi-seam mining disturbance. Under inclined conditions, the gravitational component acting along the dip direction changes the movement mode of the overlying strata, making the low side more prone to large-amplitude subsidence and compaction, whereas the high side is more susceptible to bed separation opening, sliding dislocation, and local tensile fracture propagation. In other words, movement of the overlying strata does not develop in a uniform and coordinated manner, but instead exhibits differentiated coordination characterized by low-side subsidence and compaction versus high-side tensile cracking and fracture extension. This process continuously weakens the structural integrity of the overlying strata and accelerates the bending, rotation, and unstable breakage of key strata.

The fractal evolution of fractures further reveals the internal mechanism by which the overlying strata transform from damage accumulation to instability-induced hazard development. The fractal analysis results show that the fracture fractal dimensions of the overlying strata at the M1, M2, and M3 stages are 1.023, 1.096, and 1.144, respectively, showing a continuous increasing trend. This indicates that the fracture network evolves from local discrete initiation to propagation, multiplication, and complex interconnection. With the superposition of repeated mining disturbances, fractures within the overlying strata increase simultaneously in number, scale, branching degree, and spatial connectivity. As a result, damage to the overlying strata is no longer confined to local deterioration, but gradually evolves into a complex structural system with a certain degree of connectivity. In particular, under inclined conditions, fractures on the high side are more readily controlled by tensile opening and sliding, and therefore continue to propagate, causing this zone to become the weak region and instability-sensitive area within the overlying strata structure.

Based on the integrated analysis of caving behavior, stress redistribution, displacement evolution, and numerical contact-failure patterns, the instability of the overlying strata during repeated mining of the inclined thin coal seam group can be interpreted as a coupled and progressive asymmetric failure process rather than a simple superposition of individual mining disturbances.

The first key mechanism is the repeated reconstruction of the load-bearing structure. During M1 mining, the disturbance range is limited because the mined seam is relatively thin, and the overlying strata mainly exhibit local bending subsidence and goaf compaction. During M2 and M3 mining, however, the newly induced disturbance is superimposed on the stress-relieved and structurally weakened zones formed by the previous mining stages. As a result, the overlying strata no longer respond as an intact rock mass, but as a weakened structural system with inherited deformation and accumulated damage.

The second key mechanism is inclination-controlled asymmetric load transfer. Under inclined conditions, the gravitational component along the dip direction changes the movement path of the overlying strata and causes different mechanical responses on the high side and low side. The low side tends to experience compression, compaction, and constrained deformation, whereas the high side is more prone to bed separation, sliding dislocation, tensile opening, and local contact failure. Therefore, the overlying strata do not evolve symmetrically, but form a differentiated instability pattern controlled by seam inclination.

The third key mechanism is the transformation from local disturbance to integral instability. With the upward shift of the mined seam from M1 to M3, the stress-relief zone expands, the stress concentration zone migrates, and the displacement-affected zone continuously extends upward. These processes interact with each other and gradually weaken the continuity of the load-bearing structure. Consequently, the overlying strata evolve from local subsidence and limited failure during the first mining stage to integral asymmetric subsidence and progressive structural instability under repeated mining.

Accordingly, the new insight of this study is that the hazard-causing mechanism of inclined thin coal seam group mining is not controlled by a single factor such as roof caving, stress concentration, or displacement increase alone. Instead, it is governed by the coupled evolution of repeated mining disturbance and inclination-induced asymmetric structural response. The proposed mechanism can be summarized as follows: repeated mining induces inherited stress relief and structural weakening; seam inclination controls asymmetric load transfer and movement; and their coupling promotes high-side loosening, low-side compaction, and progressive asymmetric instability of the overlying strata.

The numerical results provide useful implications for roof control, monitoring layout, and hazard prevention during repeated mining of inclined thin coal seam groups. First, the stress concentration zones ahead of the working face and near the goaf boundaries should be regarded as key areas for roof-pressure control. These zones bear transferred loads during repeated mining and may become potential locations for local roof breakage, support loading increase, or dynamic manifestations. Therefore, strengthened support, advance reinforcement, and local stress-relief measures should be preferentially considered in these regions. Second, the high-side area of the inclined coal seam group requires particular attention. The simulation results show that, under the combined influence of seam inclination and repeated mining disturbance, the high side is more prone to bed separation, sliding dislocation, and tensile or shear contact failure, whereas the low side is mainly characterized by compaction and constrained deformation. Therefore, monitoring systems should not be arranged symmetrically along the dip direction. Instead, more monitoring points should be placed on the high side, near the goaf boundary, and ahead of the working face, where stress redistribution and structural weakening are more significant. Third, the identified stress–displacement–failure coupling mechanism suggests that hazard prevention should not rely only on a single indicator. In practical mining, roof subsidence, support pressure, borehole deformation, microseismic/acoustic emission activity, and separation development should be monitored jointly. Abnormal increases in support load, rapid subsidence, or intensified microseismic activity on the high side may indicate progressive weakening of the overlying load-bearing structure. Such information can provide early warning for roof instability and guide timely adjustment of support parameters and mining sequence.

Overall, the engineering significance of this study lies in identifying the key zones and dominant processes controlling asymmetric instability of the overlying strata. The stress concentration zones ahead of the working face and near the goaf boundaries are the primary areas for load-transfer control, whereas the high-side separation and contact-failure zones are the main areas for structural-weakening monitoring. These findings can support zonal roof control, targeted monitoring layout, and prevention of mining-induced hazards in inclined coal seam group mining.

It should be noted that the numerical model in this study is mainly intended to reveal the evolution mechanism of overlying strata under repeated mining of an inclined thin coal seam group. Although the simulation results show good consistency with physical similarity simulation in terms of deformation mode, failure-range expansion, and asymmetric evolution trend, strict quantitative validation was not fully achieved because of the lack of continuous field monitoring data. Therefore, the numerical results should be interpreted as a mechanistic and trend-based analysis rather than an exact prediction of field-scale displacement or failure boundaries. Further studies should combine field monitoring data, borehole imaging, and microseismic or acoustic emission observations to improve the quantitative calibration of the model^61–64^.

## Conclusions

This study investigated the deformation, failure, and asymmetric instability mechanism of overlying strata during repeated mining of a 30° inclined thin coal seam group. The main conclusions are as follows:

(1) Repeated mining induces stagewise overburden caving and stress redistribution. During M1 mining, the overlying strata mainly exhibit local subsidence and limited failure. With the successive extraction of M2 and M3, each seam advanced to 200 m, the disturbed zone expands upward, and the stress field evolves into a zoned pattern with stress relief in the goaf and stress concentration near the working face and goaf boundaries.

(2) The displacement field shows a cumulative and asymmetric response. As mining disturbance is superimposed from M1 to M3, the high-displacement zone migrates upward and expands, and the subsidence trough deepens and widens. Controlled by the 30° inclined structure, the high side is dominated by separation and failure, whereas the low side mainly shows compaction and constrained deformation.

(3) The apparent fractal dimension of simulated fracture traces increases from 1.023 at M1 to 1.096 at M2 and 1.144 at M3, indicating a progressive increase in the relative complexity of mining-induced fracture traces.

(4) The asymmetric instability of overlying strata is controlled by the coupling of repeated mining disturbance and seam inclination. The hazard-causing process can be summarized as stress redistribution, uncoordinated subsidence, high-side fracture development, structural weakening, and asymmetric instability of the overlying load-bearing structure.

This study has several limitations. The numerical model was simplified as a two-dimensional model, and the validation mainly focused on characteristic indicators and evolution trends rather than point-by-point field calibration. In addition, 3DEC fracture images are affected by block discretization. Future work should combine field monitoring, borehole imaging, microseismic monitoring, and three-dimensional numerical modeling to further verify the proposed mechanism.

## Data Availability

All data supporting the findings of this study are available within the paper.
